# Rapid Mortality Surveillance of COVID-19 Using Verbal Autopsy

**DOI:** 10.3389/ijph.2021.1604249

**Published:** 2021-10-05

**Authors:** Amaro N. Duarte-Neto, Maria de Fátima Marinho, Lucia P. Barroso, Carmen D. Saldiva de André, Luiz Fernando F. da Silva, Marisa Dolhnikoff, Paulo Afonso de André, Catia M. Minto, Catia S. de Moura, Thábata F. Leite, Jair Theodoro Filho, Renata Aparecida de Almeida Monteiro, Philip Setel, Martin W. Bratschi, Robert Mswia, Paulo Hilário N. Saldiva, Ana Luiza Bierrenbach

**Affiliations:** ^1^ Department of Pathology, Faculty of Medicine, University of Sao Paulo, Sao Paulo, Brazil; ^2^ Vital Strategies, Sao Paulo, Brazil; ^3^ Department of Statistics, Institute of Mathematics and Statistics, University of Sao Paulo, Sao Paulo, Brazil; ^4^ State Secretary of Health of Sao Paulo, Sao Paulo, Brazil; ^5^ Vital Strategies, New York, NY, United States

**Keywords:** mortality, surveillance, COVID-like illness, minimally invasive autopsy, InterVA CRMS

Identifying causes of death, let alone COVID-19-specific mortality, is a challenge in many low- and middle-income countries. Lack of testing and large numbers of community deaths without a physician to medically certify the cause of death, are barriers to knowing the full impact of the pandemic on mortality [1].

Verbal Autopsy (VA) is a technique for determining the most medically likely causes of death in the community, where no physician is available to complete a medical certificate of cause of death. Briefly, VA uses a structured questionnaire to elicit the signs and symptoms exhibited by the deceased in the period before death and that can reliably be understood by and reported on by family members and other lay caregivers [2]. The pattern of responses to the VA questionnaire is used by physicians or a computer algorithm to assign the most probable cause of death [3, 4].

As part of the development of the Rapid Mortality Surveillance Technical Package, the WHO VA Reference Group produced a short questionnaire and algorithm, the InterVA CRMS model [5], to distinguish deaths due to COVID-like illness (CLI) from deaths due to other natural and unnatural causes. The algorithm produces estimates of the probability of death being associated with CLI, based on the answers in the short questionnaire.

This study aims to evaluate the performance of the InterVA CRMS model against ultrasound guided-minimally invasive autopsy, which is the best available reference during the pandemic. To our knowledge, no study with this purpose has been carried out yet.

## Setting

Sao Paulo is the largest city in Brazil, with more than 12 million inhabitants. It has been one of the epicenters of COVID-19, having reached in March 2021 more than 8,300 case notifications in a single day [6].

The Autopsy Service at the University of Sao Paulo (SVOC-USP) performs autopsies of natural deaths in the city of Sao Paulo for deaths without an established cause of death. On March 20th, 2020, the Governor of the State of Sao Paulo decreed emergency measures for the prevention of contagion by SARS-CoV-2, suspending conventional autopsies within the State. Since then, only a few ultrasound-guided minimally invasive autopsies (MIA-US) have been performed at the SVOC-USP.

## Approach

We applied the short questionnaire and algorithm to all the 112 deaths occurring from March to December 2020 that underwent MIA-US at the SVOC-USP.

The MIA-US procedure has been described by Duarte-Neto et al. (2020) and Dolhnikoff al. (2020) [7, 8]. Briefly, internal organs were visualized using a portable ultrasound and tissue sampling was performed using Tru-Cut^©^ semi-automatic 14G needles. Our protocol includes extensive sampling of lungs, heart, liver, kidneys, spleen, testis, skin, skeletal muscle, bone marrow, salivary glands, brain, and intestines. Reverse-transcription polymerase chain reaction (RT-PCR) was employed for molecular detection of SARS-CoV-2 in oropharyngeal swabs or pulmonary tissue as previously described [7, 9]. A team of health professionals, extensively trained in techniques appropriate to the grieving environment, asked the COVID-19 specific questions to families/caregivers after they signed the Consent Form.

## Results

According to the MIA-US, 72 deaths had COVID-19 as the cause of death (positive group), and 40 individuals died from other causes (negative group). In the positive group, 39 (54.2%) were male (mean age 54.1 ± 19.1). In the negative group, 16 (40.0%) were male (mean age 61.2 ± 21.1).

Table 1 shows the frequencies and percentages of each sign and symptom included in the short questionnaire in the positive and negative groups, and the sensitivity and specificity of each sign/symptom.

**TABLE 1:** Frequency and percentage of each sign and symptom in the groups classified as COVID-19 positive or negative by the Ultrasound-guided Minimally Invasive Autopsy - COVID-19 Case-Control study in Brazil project, Sao Paulo, Brazil, March - December 2020.

Signal/Symptom^a^	Positive n (%) (N = 72)	Negative n (%) (N = 40)	Sensitivity^b^ (95% Confidence interval)	Specificity^b^ (95% Confidence interval)
**Difficulty breathing**	—	—	91.7 (82.7–96.9)	47.5 (31.5–63.9)
yes	66 (91.7)	20 (50)	—	—
no	5 (6.9)	19 (47.5)	—	—
don´t know	1 (1.4)	1 (2.5)	—	—
**Fatigue**	—	—	79.2 (68–87.8)	42.5 (27–59)
yes	57 (79.2)	23 (57.5)	—	—
no	8 (11.1)	17 (42.5)	—	—
don´t know	7 (9.7)	0	—	—
**Fever**	—	—	75 (63.4–84.6)	82.5 (67.2–92.7)
yes	54 (75)	7 (17.5)	—	—
no	16 (22.2)	33 (82.5)	—	—
don´t know	2 (2.8)	0	—	—
**Positive test**	—	—	72.2 (60.4–95.1)	80 (64.4–90.9)
yes	52 (72.2)	0	—	—
no	7 (9.7)	32 (80)	—	—
don´t know	13 (18.1)	8 (20)	—	—
**Cough**	—	—	66.7 (54.6–77.3)	67.5 (50.9–81.4)
yes	48 (66.7)	13 (32.5)	—	—
no	23 (31.9)	27 (67.5)	—	—
don´t know	1 (1.4)	0	—	—
**Contact COVID-19**	—	—	34.7 (23.9–46.8)	80 (64.4–90.9)
yes	25 (34.7)	2 (5)	—	—
no	32 (44.4)	32 (80)	—	—
Don’t know	15 (20.8)	6 (15)	—	—
**Loss smell/taste**	—	—	18.1 (10–28.9)	80 (64.4–90.9)
yes	13 (18.1)	6 (15)	—	—
no	47 (65.3)	32 (80)	—	—
Don’t know	12 (16.7)	2 (5)	—	—

a All the answers about living in an area with social distancing/stay-at-home measures were “Yes”, those about injuries were “No”, and those about traveling to a region where COVID-19 was present were “Don't know”.

b Sensitivity was calculated here as the proportion of presence of a signal/symptom (answer “Yes”) in the group classified as positive by Ultrasound-guided Minimally Invasive Autopsy. Specificity was calculated as the proportion absence of signal/symptom (answer “No”) in the group classified as negative by Ultrasound-guided Minimally Invasive Autopsy.

**FIGURE 1:**
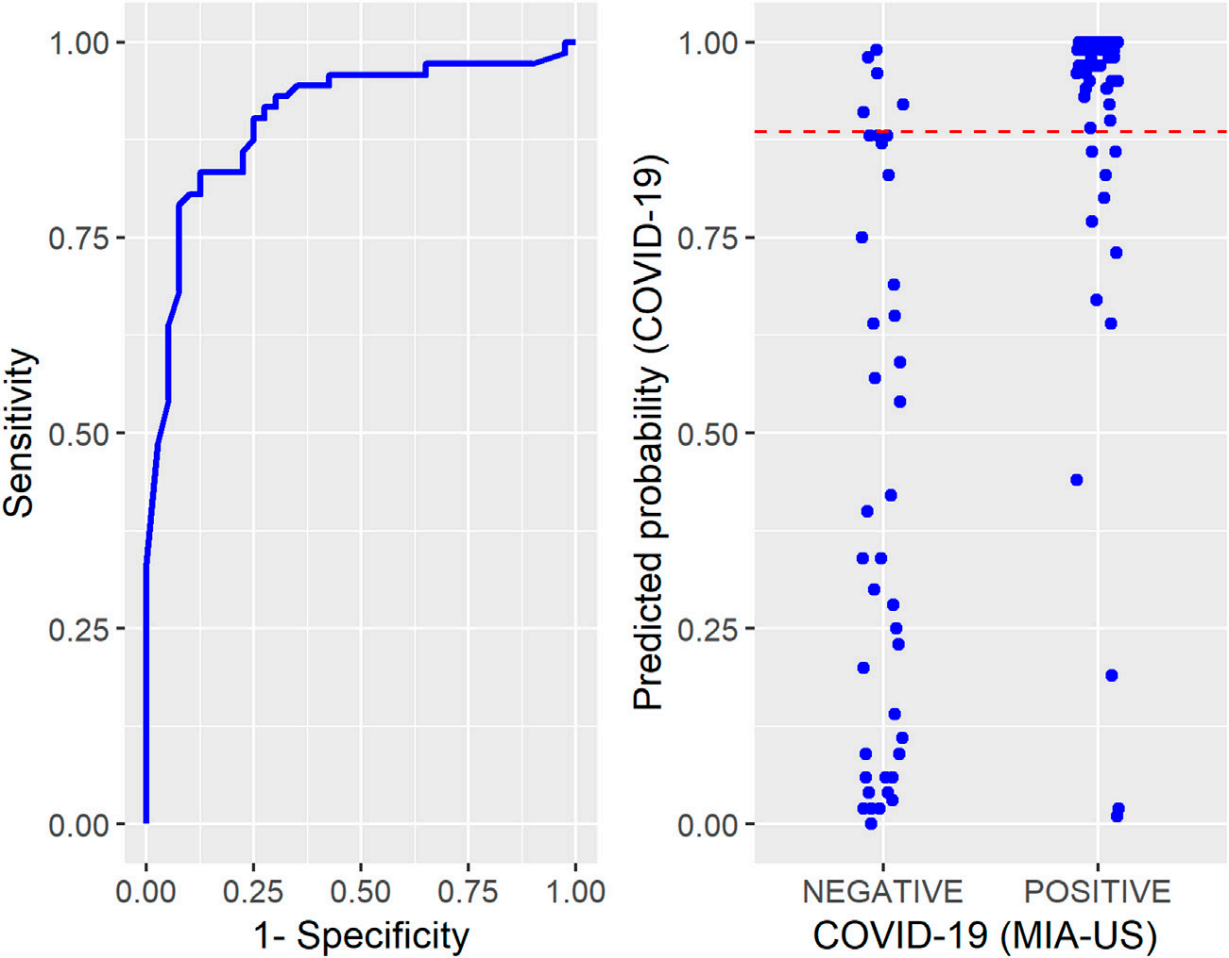
**Left:** Receiver Operating Characteristic Curve constructed to determine the cutoff for the probabilities of COVID-19 predicted by the InterVA CRMS algorithm. **Right:** Individual values of the predicted probabilities of COVID-19 according to the Ultrasound-guided Minimally Invasive Autopsy classification (positive or negative). The dashed line in red represents the cutoff - COVID-19 Case-Control study in Brazil project, Sao Paulo, Brazil, March - December 2020.

The probabilities of death by COVID-19 predicted by the InterVA CRMS algorithm (Figure 1) have high variability in the negative group while those in the positive group tend to concentrate on higher values. The cutoff value for the probability of death obtained from a ROC curve was 0.89. A sensitivity of 0.83 and a specificity of 0.88 are associated with this cutoff. The area under the ROC curve is 0.90.

MIA-US attributed COVID-19 as the cause of death to four pediatric cases, but all of them had probabilities of death due to COVID-19 smaller than the cutoff 0.89 and were classified as negative for COVID-19 by the InterVA CRMS algorithm according to the procedures described above. Excluding all cases below 20 years-old from the sample (4 pediatric cases just mentioned and one classified as negative by MIA-US), the same cutoff value was obtained from the ROC curve (area under curve = 0.93) and this was associated with a sensitivity of 0.88 and a specificity of 0.87.

## Discussion

Our results show that InterVA CRMS is a reliable way to rapidly track mortality due to CLI in adults with high sensitivity and specificity. The application of a questionnaire like this, quick and easy to understand, followed by the use of an algorithm for reading the results, which requires minimum computer capacity, can help to count cases of death by CLI in communities without extensive testing and medical certification of cause of death. In addition, initiatives such as this have their role even in large urban centers with well-established autopsy services, since most of them have had their activities disrupted during the pandemic due to the risk of contagion of staff members.

Adjustments to the questionnaire and algorithm are likely to be needed, not only to better discriminate the disease among children, but also as the epidemic progresses and as more knowledge is accrued. For example, the inclusion of a question of whether the decedent had been vaccinated for COVID-19 would be helpful for the cause of death assignment. Once large and representative samples of InterVA CRMS data with vaccination status are made available, they could be used to assess fatal vaccine failure.

## Conclusion

Although more validation studies are needed, our findings indicate that COVID-19 deaths can be correctly assigned in adults using a simple set of questions about their signs and symptoms, helping in directing control measures during the course of the pandemic.
